# The Efficacy and Safety of a Qiliqiangxin Capsule Combined with Sacubitril/Valsartan in the Treatment of Chronic Heart Failure: A Systematic Review and Meta-Analysis

**DOI:** 10.1155/2023/2701314

**Published:** 2023-02-03

**Authors:** Wensheng Chen, Huihua Chang, Xiaoqi Wang, Yuanping Wang, Yuntao Liu, Dawei Wang

**Affiliations:** ^1^Guangdong Provincial Hospital of Chinese Medicine, The Second Affiliated Hospital of Guangzhou University of Chinese Medicine, Guangzhou 510120, China; ^2^Shunde Hospital of Guangzhou University of Chinese Medicine, Guangzhou University of Chinese Medicine, Guangzhou 528333, Guangdong, China; ^3^The First Affiliated Hospital, Guangzhou University of Chinese Medicine, Guangzhou 510407, China

## Abstract

**Background:**

Qiliqiangxin (QLQX) capsules are a commonly used proprietary Chinese medicine for the adjuvant treatment of chronic heart failure (CHF) in China. In recent years, several randomized controlled trials (RCTs) have reported on the efficacy and safety of QLQX combined with sacubitril/valsartan for CHF.

**Objective:**

The purpose of this study was to systematically analyze the clinical efficacy and safety of QLQX combined with sacubitril/valsartan in the management of CHF and to provide clinicians as well as scientists with optimal evidence-based medical evidence.

**Methods:**

We searched RCTs to evaluate the efficacy and safety of QLQX combined with sacubitril/valsartan in the treatment of CHF in the Wanfang Database, China National Knowledge Infrastructure, China Science and Technology Journal Database, PubMed, Embase, and Cochrane Library databases from their inception until January 8, 2022. RCTs on QLQX in combination with sacubitril/valsartan for CHF were included. The outcome measures considered were total effective rate, left ventricular ejection fraction (LVEF), left ventricular end-diastolic dimension (LVEDD), 6-minute walking distance (6-MWD), and adverse events. The quality of the included RCTs was assessed thereafter using the Cochrane risk of bias tool. RevMan 5.3 software was used to conduct the meta-analysis.

**Results:**

The meta-analysis included 17 trials involving 1427 CHF patients. The results indicated that with sacubitril/valsartan administration combined with QLQX treatment, the total effective rate (relative risk (RR) = 1.24; 95% confidence interval (CI) (1.17, 1.31); *p* < 0.01), LVEF (mean difference (MD) = 6.20; 95% CI (5.36, 7.05; *p* < 0.01)), and 6-MWD (MD = 55.87; 95% CI (40.66, 71.09); *p* < 0.01) of CHF patients were significantly increased, and the LVEDD value of CHF patients was noted to be significantly reduced (MD = −3.98; 95% CI (−4.47, −3.48); *p* < 0.01). Moreover, there was no increase in the number of adverse events during treatment (RR = 0.67; 95% CI (0.33, 1.34); *p* < 0.01).

**Conclusions:**

This study indicated that in CHF patients, on the basis of sacubitril/valsartan treatment, combination with QLQX can potentially enhance the total effective rate, improve LVEF and 6-MWD, and reduce LVEDD values, with good safety. However, considering the poor quality of the included studies, a multicenter, randomized, double-blind controlled study is needed for further confirmation.

## 1. Introduction

Chronic heart failure (CHF), which is the end-stage manifestation of cardiovascular disease and the leading cause of mortality, is one of the two major challenges in the cardiovascular field in the 21st century [1]. Globally, approximately 2% of the population suffers from CHF, and the prevalence is continuously increasing [2]. The absolute number of patients with CHF has also been increasing due to an aging population, global population growth, and improved survival rates after the initial diagnosis [3]. The magnitude of this public health issue can be reflected in the heavy economic burden of a CHF-related health expenditure of approximately $31 billion per annum, which is expected to increase to $70 billion by 2030 [4]. In recent years, several new drugs have been used to treat CHF. Sacubitril/valsartan is a novel orally active angiotensin receptor-enkephalinase inhibitor. A single cocrystal composed of batroxobin and valsartan at a molar ratio of 1 : 1 has been approved as the first-line drug for the treatment of heart failure [5]. Several lines of evidence demonstrate that sacubitril/valsartan can significantly reduce the risk of cardiovascular death and hospitalization in heart failure patients [6]. Despite advances in sacubitril/valsartan therapy, however, only 20–40% of heart failure patients with reduced ejection fraction have been found to be eligible for initiation therapy with sacubitril/valsartan [7]. CHF still displays a high mortality rate and a poor quality of life; therefore, the development of new drugs with which to treat CHF remains an urgent issue [8]. Chinese herbal medicines have been found to serve as important sources of potential drugs for the treatment of heart failure [9]. In China, to improve the clinical efficacy and safety of sacubitril/valsartan and expand its application, doctors often combine sacubitril/valsartan with Chinese herbal medicines to effectively treat CHF [10].

The Qiliqiangxin (QLQX) capsule, a well-known proprietary Chinese medicine, has been approved by the National Medical Products Administration and is widely used as an adjunctive therapy in the treatment of CHF. QLQX consists of 11 different herbs: *Astragalus mongholicus* Bunge (Fabaceae), *Panax ginseng* (C. A. Mey.) (Araliaceae), *Aconitum lethale Griff.* (Ranunculaceae), *Salvia miltiorrhiza Bunge* (Lamiaceae), *Descurainia Sophia* (L.) *Webb ex prantl* (Brassicaceae), *Alisma plantago-aquatica subsp. orientale* (Sam.) Sam. (Alismataceae), *Polygonatum odoratum* (Mill.) Druce (Asparagaceae), *Neolitsea cassia* (L.) Kosterm (Lauraceae), *Carthamus tinctorius* (L.) (Asteraceae), *Periploca sepium Bunge* (Apocynaceae), and *Citrus aurantium* (L.) (Rutaceae). In addition, various preclinical studies have shown that QLQX can significantly inhibit pathological cardiac remodeling through a multitargeted, multipathway mechanism of action. For example, it has been previously shown that QLQX can exert its antiapoptotic effects through the activation of PPAR gamma, thereby reducing pathological hypertrophy and improving myocardial fibrosis in isoproterenol-induced CHF mice [11]. Interestingly, in a left anterior descending coronary artery ligation-induced CHF mouse model, QLQX was also observed to markedly improve cardiac function, myocardial fibrosis, and inflammatory infiltration in rats with heart failure, which may be related to the optimization of glucose metabolism in both the distal and border zone myocardium by QLQX [12]. In addition, in a clinical study involving a multicenter, randomized, double-blind controlled study [13], QLQX was found to substantially improve cardiac function, increase the left ventricular ejection fraction (LVEF), increase the 6-minute walking distance (6-MWD), and thereby reduce composite endpoint events in patients with CHF with a favorable safety profile. All of these studies have suggested that QLQX can serve as a promising potential therapeutic agent for the treatment of CHF. In the last 5 years, clinical studies conducted on QLQX combined with sacubitril/valsartan for the treatment of CHF have been increasing, and these clinical studies have indicated that the combination of QLQX and sacubitril/valsartan may not only exhibit improved clinical efficacy in treating CHF but also reduce the incidence of various adverse events [14, 15]. However, individual studies have been inconsistent, which may be due to their limitations. Therefore, we conducted a meta-analysis to evaluate the potential efficacy and safety of QLQX combined with sacubitril/valsartan in the treatment of CHF.

## 2. Methods

### 2.1. Protocol and Registration

Before carrying out this study, we registered our research proposal on the website of Open Science Framework at the following registered address: https://osf.io/mkswr. This study followed a PRISMA (Preferred Reporting Items for Systematic Reviews and Meta-Analyses) statement [16] as shown in Supplementary Material S1.

### 2.2. Search Strategies

Two reviewers independently searched three Chinese electronic databases, namely, the Wanfang Database, China National Knowledge Infrastructure, and China Science and Technology Journal Database, and three English electronic databases, namely, PubMed, Embase, and the Cochrane Library. In addition, to find various unpublished clinical studies, we also searched clinicaltrials.gov and the Chinese Clinical Trial Register (ChiCTR) website to further explore the different ongoing clinical trials. Our search keywords were “heart failure,” “Qiliqiangxin,” and “sacubitril/valsartan,” and the search time was from inception to January 8, 2022. We also manually searched the reference lists of all full-text papers for other relevant reports.

### 2.3. Inclusion Criteria

#### 2.3.1. Type of Studies

Randomized controlled trials (RCTs) that investigated the effectiveness and safety of QLQX for CHF were included.

#### 2.3.2. Type of Participants

The participants met the recognized diagnostic criteria, such as the “2016 ESC Guidelines for the diagnosis and treatment of acute and chronic heart failure” [17] and the “Guidelines for diagnosis and treatment of heart failure in China 2018” [18]. Race, sex, and source of the case were not limited.

#### 2.3.3. Types of Interventions

The control group should have been treated with a conventional treatment regimen (e.g., diuretics, beta-blockers, and aldosterone antagonist) in combination with sacubitril/valsartan, and the intervention group should have been treated with QLQX capsule on the basis of the control group, without limiting the dose, frequency, duration of treatment, or manufacturer of QLQX.

#### 2.3.4. Types of Outcome Measures

The outcome indicators were the LVEF, left ventricular end-diastolic dimension (LVEDD), 6-MWD, and total effective rate, and treatments resulting in remission of the various clinical signs and symptoms of CHF and with cardiac function improvement at grade 1 or above after treatment were considered to be “effective” according to the NYHA scale; otherwise, they were considered to be “ineffective.” The ratio of the number of effective cases to the total number of cases was considered to be the effective rate.

### 2.4. Safety Outcome

All adverse events that occur after a subject has received a test dose may be manifested by the presence of symptoms, signs, disease, or abnormal laboratory tests.

### 2.5. Exclusion Criteria

Exclusion criteria include the following:

- Case reports, literature reviews, meta-analyses, conference papers, and so on
- Inability of the study outcomes to be extracted
- Animal experiments

### 2.6. Study Selection and Data Extraction

The literature selection and data extraction were carried out by two reviewers independently. If there was any disagreement during this process, it was decided primarily by means of discussion or seeking a third reviewer. The reviewers first read the title and abstract of the article, screened out the literature that could be included according to the inclusion criteria, further obtained the full text, and then read the full-text information in detail to screen the literature that accurately met the criteria. The data extraction was mainly performed to extract basic data from the included studies, such as the first author, year of publication, participation, mean age, sex, intervention measures with specific drugs (such as QLQX dose, frequency, and manufacturer), treatment time, follow-up time, funding for information, posttreatment follow-up, clinical effectiveness of the CHF patients' LVEF before and after the treatment, potential changes in LVEDD and 6-MWD, and associated adverse events. If missing information was found during data extraction, the authors of the original study were contacted via e-mail to obtain it.

### 2.7. Risk of Bias Assessment

In this study, two researchers independently assessed the quality of the included trials according to the Cochrane bias risk assessment tool [19]. The assessment was carried out by means of evaluation of the following seven different items: (1) random sequence generation, (2) allocation concealment, (3) blinding of participants and personnel, (4) blinding of outcome assessment, (5) incomplete outcome data, (6) selective reporting, and (7) other bias. Each item was potentially assessed as having a high risk, low risk, or unclear risk.

### 2.8. Statistical Synthesis and Analysis

In this study, RevMan v.5.3 software was used for meta-analysis of the relevant outcome indicators, and the effective values of dichotomous variables were expressed as the relative risk (RR) and the 95% confidence interval (CI). Continuous variables were represented by the mean difference (MD) and the 95% CI, and *p* < 0.05 indicated a significant difference. In this study, the chi-square test was also used to test the heterogeneity of the included literature, and appropriate statistical methods were selected to analyze the results according to the heterogeneity [20]. If *I*^2^ was 0, this meant that there was relatively little possibility of heterogeneity among the multiple similar studies and that the homogeneity was good. If *I*^2^ was <50%, this indicated small interstudy heterogeneity and that a fixed-effects model could be used for statistical analysis; if *I*^2^ was >50%, this indicated large interstudy heterogeneity, and the possible source of heterogeneity was analyzed first; if it was determined that the various clinical factors caused large heterogeneity, a subgroup analysis was also performed. However, if after the subgroup analysis the heterogeneity was still large, a random-effects model was selected to calculate the statistics.

### 2.9. Subgroup Analysis

Considering that the age of the patients and the duration of the treatment will affect the efficacy of QLQX in the treatment of CHF, we conducted a subgroup analysis based on the aforementioned two factors.

### 2.10. Sensitivity Analysis

Sensitivity analysis is a common analytical method employed to quantitatively describe the influence of the corresponding input variables of a specific model upon its corresponding output variables. According to the scope of its various effects, it can be divided into global and local sensitivity analyses. This study mainly used local sensitivity analysis (conducted by the package “meta” (version 5.2-0) in *R* (version 4.1.2)). The main method comprised removing each included RCT from the meta-analysis and then again conducting a comprehensive statistical analysis, which involved redrawing the forest map of the meta-analysis, followed by comparing the results with those of previous studies to evaluate the stability of the results [21].

### 2.11. Publication Bias

If the observed outcome indicators were more than 10 included studies, a funnel plot was used to evaluate publication bias. However, if the funnel plot was asymmetric, Egger's test (conducted by the package “meta” (version 5.2-0) in *R* (version 4.1.2)) was employed to further evaluate the degree of publication bias, and *p* < 0.05 was considered to indicate the existence of publication bias.

## 3. Results

### 3.1. Search Results

**Figure 1:**
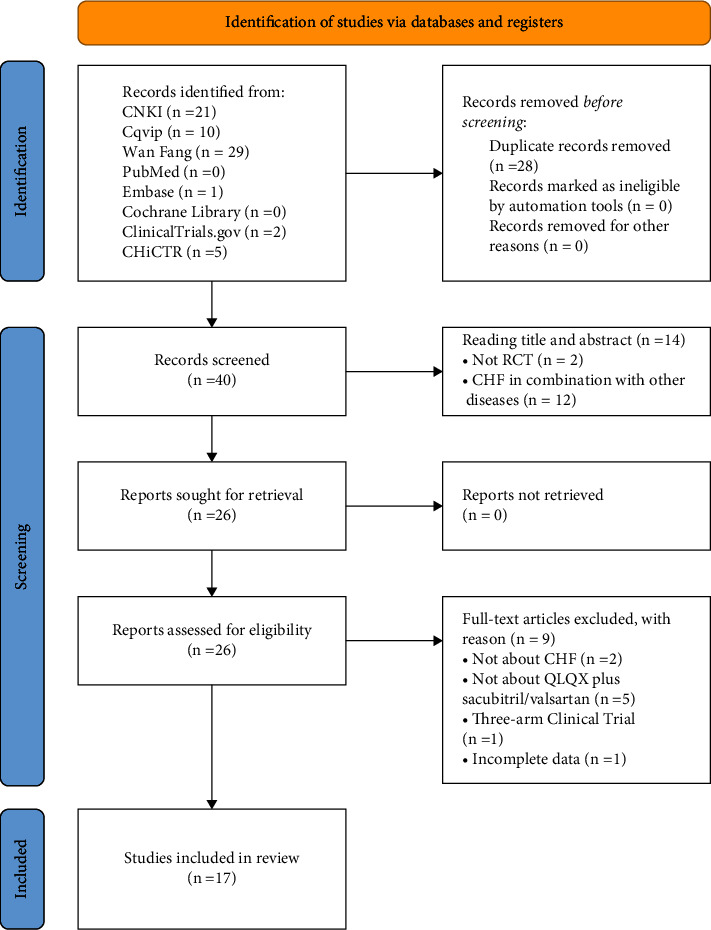
PRISMA flow diagram.

A total of 68 studies were retrieved from the aforementioned database, leaving a total of 40 studies after removing the various duplicates. After reading the title and abstract, 14 studies were excluded because 12 of them included patients suffering from other diseases (e.g., atrial fibrillation and coronary heart disease), and two of them were non-RCT studies. The full text of the 26 studies was further obtained. After reading the full text, nine studies were excluded, including two studies whose subjects were defined as having cardiac insufficiency and did not meet the diagnostic criteria for CHF and one study that was also excluded due to the use of a multiarm clinical trial. In addition, since the duration of the treatment was not reported in one study, we also contacted the corresponding author of the article via e-mail to obtain more detailed information but failed to do so. Thus, considering the incompleteness of the study, this paper was excluded from the meta-analysis. The remaining five studies were excluded from inclusion because the interventions were based on the QLQX only. Finally, only 17 studies [14, 15, 22–36] were included for systematic evaluation and meta-analysis. A flowchart of the study selection is shown in Figure 1.

### 3.2. Study Characteristics

The 17 studies [14, 15, 22–36] involved a total of 1427 patients (712 in the intervention group and 715 in the control group). The sample sizes ranged from 40 to 120. The mean age ranged from 35.2 to 76.5 years. The control group was treated with sacubitril/valsartan in addition to the conventional treatment. Meanwhile, the intervention group was treated with QLQX capsules on the basis of the control group. QLQX was obtained from Shijiazhuang Yiling Pharmaceutical Co., Ltd. (Shijiazhuang, China). The treatment lasted for a duration of 28 to 90 days. However, none of the studies involved follow-up with patients. Five studies [24–26, 28, 29] were supported by national funding. The details of all studies are summarized in Table 1.

### 3.3. Quality Evaluation

The Cochrane bias risk assessment tool was used to assess the quality of each study. We tried to e-mail the corresponding author of the article to collect more information on these selected studies; unfortunately, we did not receive any response. Fourteen trials [14, 15, 22–27, 29–32, 35, 36] were found to report a random allocation of participants, but only seven trials [14, 24–27, 31, 32] mentioned the use of a random number table. One study [33] used the Excel random sampling method for the grouping, which was considered to have a “low risk,” while the remaining six trials [15, 22, 23, 29, 35, 36] did not mention the method used for randomization in the sequence generation, which were therefore selected as having a “high risk.” In addition, one trial [34] performed the grouping according to the therapeutic agent administered, which was considered to have a “high risk.” The remaining two trials [28, 33] did not mention randomized grouping; therefore, we were not sure of the exact grouping method used and, thus, these were judged to have an “high risk.” In terms of the allocation concealment, none of the studies mentioned specific information on allocation concealment and, therefore, were judged to have an “unclear risk.” Regarding the implementation of blinding, none of the studies were blinded to the patients, study personnel, and outcome measures; therefore, these features were judged to have a “high risk.” In addition, all of the included RCTs contained no incomplete data or selective reporting and were labeled as having a “low risk.” Although all of the included studies reported consistent findings regarding between-group baselines, potential sources of bias (such as intention to treat and other adherence differences) may still exist. Therefore, other biases were assessed as having an “unclear risk” after attempts made to contact the authors via e-mail to clarify the various unreported information but did not receive a response. The results of the risk of bias assessment are shown in Table 2.

### 3.4. Outcomes

#### 3.4.1. Total Effective Rate

**Figure 2:**
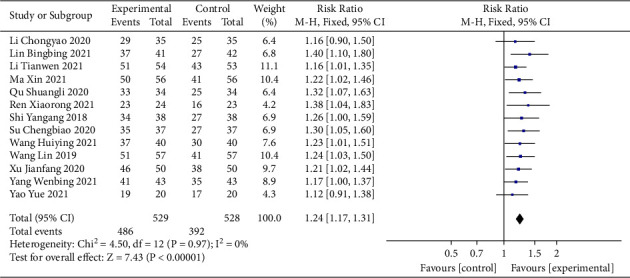
Forest plot of total effective rate.

Thirteen studies [15, 22, 24–33, 35] involving 1057 participants reported the total effective rate. The heterogeneity test results indicated that the heterogeneity of the studies among each group was small (*I*^2^ = 0%), and the fixed-effects model was used for the meta-analysis. The results showed that therapy involving sacubitril/valsartan combined with QLQX significantly improved the total effective rate of CHF patients (RR = 1.24; 95% CI (1.17, 1.31); *p* < 0.01; Figure 2). Sensitivity analysis showed that the results were robust (Supplementary Material S2.1). In addition, subgroup analysis was performed to consider differences in the treatment duration and the age of included patients between the study groups. The results of the subgroup analysis showed that QLQX combined with sacubitril/valsartan was superior to sacubitril/valsartan alone in improving the total effective rate, regardless of the age of CHF patients being ≥65 years or <65 years and regardless of the duration of treatment being >30 days or ≤30 days. Moreover, subgroup analysis according to different ages and treatment durations showed no significant difference between the groups (*p* for interaction = 0.66 and 0.73, respectively; Supplementary Materials S2.2 and S2.3).

#### 3.4.2. LVEF

**Figure 3:**
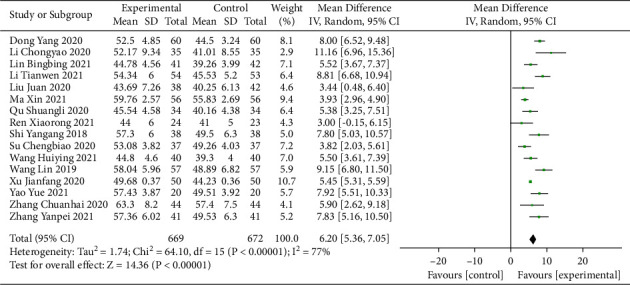
Forest plot of LVEF.

Sixteen studies [14, 15, 22–31, 33–36] involving 1341 participants reported the LVEF. The results of the heterogeneity test showed that the heterogeneity of the studies among each group was relatively high (*I*^2^ = 77%). Sensitivity analysis conducted by omitting studies one by one did not reveal significant changes in the heterogeneity and the pooled effect, and the random-effects model was used for the meta-analysis (Supplementary Material 2.4). The results suggested that therapy involving sacubitril/valsartan combined with QLQX significantly improved the LVEF value of CHF patients (MD = 6.20; 95% CI (5.36, 7.05); *p* < 0.01; Figure 3). Subgroup analysis according to different ages and treatment durations showed no significant difference between the groups (*p* for interaction = 0.11 and 0.94, respectively; Supplementary Materials S2.5 and S2.6).

#### 3.4.3. LVEDD

**Figure 4:**
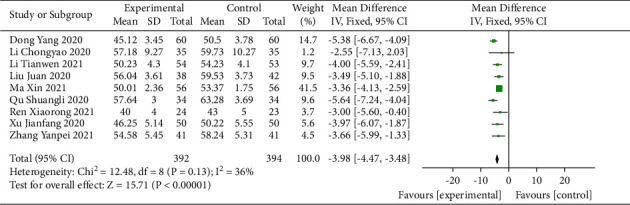
Forest plot of LVEDD.

Nine studies [14, 22–25, 27, 28, 31, 34] involving 786 participants reported the LVEDD. The results of the heterogeneity test indicated that the heterogeneity of the studies between the groups was relatively small (*I*^2^ = 36%), and a fixed-effects model was used for the meta-analysis. The results showed that with sacubitril/valsartan administration combined with QLQX treatment, the LVEDD value of the CHF patients was significantly reduced (MD = −3.98; 95% CI (−4.47, −3.48); *p* < 0.01; Figure 4). Sensitivity analysis showed that the results were robust (Supplementary Material S2.7). Subgroup analysis according to different ages and treatment durations showed no significant difference between the groups (*p* for interaction = 0.52 and 0.35, respectively; Supplementary Materials S2.8 and S2.9).

#### 3.4.4. 6-MWD

**Figure 5:**
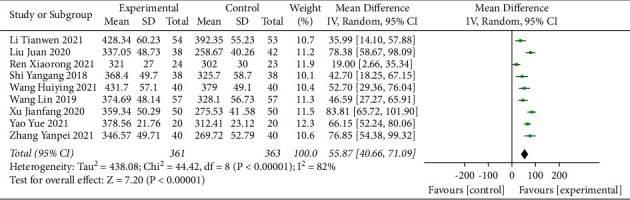
Forest plot of 6-MWD.

Ten studies [15, 23, 24, 28, 30, 31, 33–36] reported the 6-MWD outcome. The results of the heterogeneity test showed that the heterogeneity of the studies between the various groups was significantly high (*I*^2^ = 99%). Sensitivity analysis was performed by excluding studies one by one. After removing the studies reported by Zhang [36], the heterogeneity between studies was significantly reduced (*I*^2^ = 82%; Supplementary Material S2.10). However, the heterogeneity was still >50%, and a random-effects model was used for the meta-analysis. The results indicated that treatment involving sacubitril/valsartan combined with QLQX significantly increased the 6-MWD of CHF patients (MD = 55.87; 95% CI (40.66, 71.09); *p* < 0.01; Figure 5). Subgroup analysis according to different ages and treatment durations showed no significant difference between the groups (*p* for interaction = 0.38 and 0.48, respectively; Supplementary Materials S2.11 and S2.12), thereby suggesting that age and treatment duration may not be the primary sources of the heterogeneity.

### 3.5. Adverse Events

**Figure 6:**
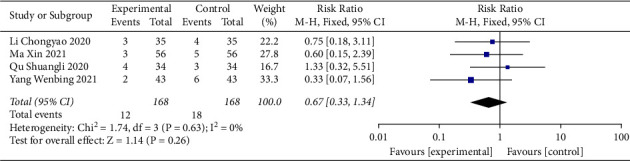
Forest plot of adverse events.

Of the 17 included studies, eight trials [14, 15, 23, 28, 31, 33, 34, 36] did not report adverse events. Adverse events were reported in nine studies [22, 24–27, 29, 30, 32, 35], among which no adverse reactions were observed during treatment in five studies [24, 26, 29, 30, 35], and some adverse reactions (such as nausea, vomiting, headache, and dizziness) were observed in both the intervention group and the control group during treatment in four studies [22, 25, 27, 32]; there were 3.4% (12/358) adverse reactions in the intervention group and 4.7% (17/358) adverse reactions in the control group, as shown in Table 3. The results of the heterogeneity test showed that the heterogeneity of the various studies between the groups was relatively small (*I*^2^ = 0%), and a fixed-effects model was used for the meta-analysis. The results indicated that for sacubitril/valsartan combined with QLQX, there was no significant increase in adverse events during the treatment period (RR = 0.67; 95% CI (0.33, 1.34); *p* < 0.01; Figure 6).

### 3.6. Publication Bias

**Figure 7:**
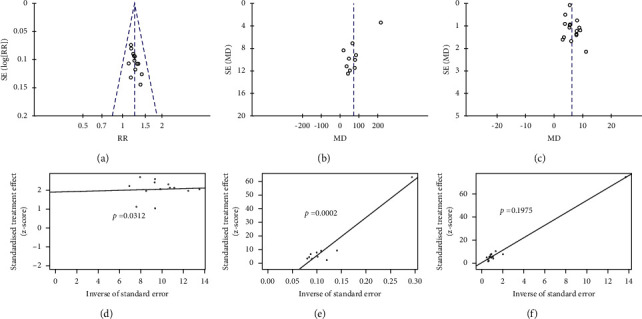
Funnel plot of the (a) total effective rate, (b) 6-MWD, and (c) LVEF and Egger's test of the (d) total effective rate, (e) 6-MWD, and (f) LVEF.

The RCTs of the total effective rate, LVEF, and 6-MWD were found to be 13, 16, and 10, respectively, which were significantly greater than 10, and funnel plots were constructed to assess the potential publication bias. A funnel plot of data relating to the total effective rate and 6-MWD showed asymmetry (Figures 7(a) and 7(b)), and Egger's test (Figures 7(d) and 7(e)) showed statistical significance (*p*=0.0312 and 0.0002). A funnel plot of the LVEF data showed symmetry (Figure 7(c)), and the result of Egger's test (Figure 7(f)) showed no statistical significance (*p*=0.1975).

## 4. Discussion

### 4.1. Main Results of This Research

In this study, we conducted a comprehensive search of the various relevant clinical randomized studies of QLQX combined with sacubitril/valsartan for the treatment of CHF by searching relevant databases in both English and Chinese, which resulted in a total of 17 studies involving 1427 participants for meta-analysis. Our study showed that QLQX combined with sacubitril/valsartan performed significantly better in terms of improved overall efficacy, LVEF, and 6-MWD and reduced LVEDD than did sacubitril/valsartan alone. The results did not change in the subgroup analysis based on age and treatment duration. Regarding the safety of therapy with sacubitril/valsartan combined with QLQX, there was no significant increase in adverse events during the treatment period, and no serious adverse events were observed in either group, which suggested that QLQX was generally effective and safe for patients with CHF.

We analyzed the quality of all the included studies through the Cochrane bias risk assessment tool, and our results indicated that the quality of all the included studies was relatively low. First, in terms of the randomization method, most of the included original literature only stated that “randomized” was used. Moreover, in terms of the randomization method, most of the included original literature only stated the word “random” but did not accurately describe the specific randomization method used, or the randomization method employed was not reasonable. Second, in terms of allocation concealment, none of the included studies described the allocation concealment. In addition, in terms of blinding implementation, none of the included studies were blinded to the patients, investigators, or statisticians. Finally, none of the studies registered their study protocols in clinical research registries (e.g., the Chinese Clinical Trial Registry) at the time at which they were conducted. These results suggested that the poor quality of our included studies could further significantly reduce the credibility of the results of our meta-analysis. Based on the shortcomings of the currently available clinical studies related to the use of QLQX in combination with sacubitril/valsartan for the treatment of CHF, we make the following recommendations for conducting future clinical studies. First, researchers should refer to the contents of the CONSORT statement [37] in detail, develop a proper study protocol, and register it on the relevant websites before conducting the clinical studies. Second, an appropriate random assignment method (e.g., random number table) should be selected to assign the subjects to different groups, with detailed descriptions of the hidden contents of the assignment. In addition, if possible, the studies should be conducted in a blind manner, and subjects as well as the control group can be placebo-controlled, which would potentially exclude the placebo effect of QLQX.

The total effective rate is a comprehensive index for assessing significant improvement in patients with heart failure and is widely used as a review indicator for the various drugs used to treat heart failure [26]. Our meta-analysis showed that QLQX combined with sacubitril/valsartan was substantially better than sacubitril/valsartan alone in improving the treatment efficiency in CHF patients. Moreover, subgroup analysis showed no significant effect of the treatment duration and the mean age of the participants on this outcome, with the good heterogeneity and the sensitivity analysis suggesting robust results.

We also observed significant improvements in LVEF, LVEDD, and 6-MWD values following the use of QLQX combined with sacubitril/valsartan for CHF. LVEF and LVEDD are considered to be important indicators of cardiac function and play an important role in the prognosis of heart failure patients [38, 39]. The 6-min walk test [40] is a simple, safe, and convenient test that has been widely used to assess the exercise tolerance of patients with CHF and evaluate the severity of heart failure. Our results suggested that QLQX combined with sacubitril/valsartan was superior to sacubitril/valsartan alone in improving LVEF and 6-MWD and, thus, reducing LVEDD in patients with CHF. However, we also observed greater heterogeneity in the two outcome indicators (LVEF and 6-MWD). Therefore, we performed a meta-analysis by means of a random-effects model; in addition, we conducted a subgroup analysis based on the duration of the treatment and the mean age of the participants included in the study. We observed that none of the meta-analysis results were reversed, but the heterogeneity was also not reduced, and the sensitivity analysis suggested robust results. Furthermore, upon reviewing the characteristics of the included studies, we found that the source of heterogeneity may potentially arise from the fact that various aspects of the routine treatment of the participants differed between studies. In addition, all included clinical studies were conducted primarily within Chinese hospitals, most of which are at the county level in China, with varying levels of care and significant variation in the standardization of medication for CHF, further increasing the clinical heterogeneity between studies.

It has been established that the treatment goals for CHF include improving the quality of life, improving long-term prognoses, and reducing morbidity, mortality, and hospitalization rates [41]. However, only a few included studies focused on the various outcome indicators to improve quality of life, such as the Kansas City Cardiomyopathy Quality of Life Questionnaire [42] and the Minnesota Heart Failure Quality of Life Questionnaire [43]. Moreover, none of the studies have undertaken long-term follow-ups with patients. Reductions in both mortality and hospitalization rates are also considered to be key indicators with regard to the evaluation of heart failure medications [41], which, unfortunately, were not observed in any of the reported studies. In conclusion, it is still unknown whether QLQX combined with sacubitril/valsartan can improve the quality of life of patients with heart failure, improve long-term prognoses, and reduce mortality and hospitalization rates; therefore, future clinical studies should focus on these important outcome indicators.

In addition to clinical efficacy, safety is also regarded as the most important concern in herbal formulations. In the present study, regarding safety, we observed that, in comparison with sacubitril/valsartan alone, QLQX combined with sacubitril/valsartan did not show significant adverse effects during the treatment of CHF, which was consistent with the findings of various previous studies. This finding appeared to indicate that QLQX is relatively safe in the treatment of CHF, but we should also clearly recognize that there were eight studies [14, 15, 23, 28, 31, 33, 34, 36] that did not report their safety results; therefore, the safety profile of QLQX was unclear in these studies. In addition, the long-term clinical safety of QLQX is unknown due to the lack of long-term follow-up data. In future studies, we should also observe the long-term safety profile of QLQX during the treatment of CHF.

The pharmacokinetic study of compound traditional Chinese medicine is the core of the modernization of traditional Chinese medicine, which can provide an experimental basis for rational clinical drug use and safety evaluation [44]. A previous pharmacokinetic study used high-performance liquid chromatography-tandem mass spectrometry (HPLC–MS/MS) technology to screen the main active ingredients of QLQX [45]. The research results showed that there were mainly 29 active ingredients of QLQX: 10 of these were saponins (astragaloside, ginsenoside Rg1, Rg3, Rb1, Rb2, Rc, Rd, Re, Rf, and F2), six were phenolic acids (salvianolic acid A, salvianolic acid B, lithospermic acid, danshensu, rosmarinic acid, and protocatechuic acid), six were flavonoids (calycosin-7-glucoside, formononetin, hesperidin, hydroxysafflor yellow A, rutin, and quercetin), and seven were alkaloids (aconitine, hypaconitine, mesaconitine, benzoylaconine, benzoylhypaconine, enzoylmesaconine, and sinapine bisulfate) [45]. Some active ingredients have been shown to have protective effects on the heart. For example, astragaloside can inhibit ventricular remodeling and improve cardiac function by reducing oxidative stress in cardiomyocytes and reducing mitochondrial damage [46], and salvianolic acid may exert cardioprotective effects by promoting angiogenesis [47].

### 4.2. Publication Bias

Our study also showed publication bias for the outcome measures of the total effective rate and 6-MWD, which was inevitable because all of the studies were based in China and reported positive results. Although we searched the current major clinical research registries in an attempt to include various unpublished studies, we did not find any relevant studies. Thus, considering that none of the included original studies were registered prior to trial initiation, we suggest that future investigators follow the clinical trial registration system to bring their clinical trials into the public domain from the very beginning, increase the transparency of the clinical trials, and improve the overall standard of the clinical trials, thereby effectively reducing the publication bias.

### 4.3. Comparison with Other Meta-Analyses

In this study, we compared the findings of various previously reported meta-analyses. For instance, Xu's et al. [10] research team systematically evaluated the clinical efficacy and safety of QLQX combined with conventional drugs in the treatment of CHF, and the findings indicated that the combination of QLQX with conventional drugs was not limited to QLQX combined with sacubitril/valsartan. Their subgroup analysis showed that QLQX combined with sacubitril/valsartan was significantly better than sacubitril/valsartan alone in improving clinical efficiency and LVEF, which was also a finding that was consistent with the present study, but the previous report only included four clinical trials, totaling 358 patients with a search deadline of August 2020. In contrast, the present study included 17 clinical trials with a total of 1427 patients with CHF, and our current meta-analysis is the most recent and comprehensive, which further strengthens the results of the previous meta-analysis. In addition, our study is more reproducible because we detail the screening details of the literature in the appendix.

### 4.4. Limitations

There are some limitations associated with this study that are worth discussing. First, we found large heterogeneity in the assessment of the effect of QLQX combined with sacubitril/valsartan in improving LVEF and 6-MWD in patients with CHF, as indicated by the *I*^2^ values. This is predictable because of the interstudy differences in the quality of studies evaluated, the populations enrolled, and the specific dosing regimens of treatment (e.g., conventional drug therapy). Despite our use of random-effects models and subgroup analysis, the study's heterogeneity persisted. Second, we conducted only a quantitative meta-analysis based primarily on the secondary data, which might have led to inaccurate results due to insufficient raw individual patient data. Furthermore, considering that Chinese herbal medicines are also popularly used in Asian countries such as South Korea and Japan and given that only Chinese and English databases were searched in this study, it is possible, albeit highly unlikely, that other relevant literature was missed during analysis.

## 5. Conclusion

Overall, our study demonstrated a significantly better efficacy of QLQX combined with sacubitril/valsartan in comparison with sacubitril/valsartan alone in terms of an improved total effective rate, increased LVEF and 6-MWD, and reduced LVEDD with a favorable safety profile in the CHF treatment. However, because of the poor quality of the original study, more high-quality clinical studies are needed to verify the clinical efficacy and safety of QLQX.

## Figures and Tables

**Table 1:** Basic characteristics of included studies.

Study	Sample size I/C	Age (years) I/C	Sex (M/F)	Treatment	Comparator	Follow-up	Treatment duration (days)	End points	Funding
Dong and Du 2020	60/60	68.9 ± 4.56/71.3 ± 5.62	I: 37/23	Sacubitril/valsartan plus QLQX 1.2 g, tid + conventional treatment	Sacubitril/valsartan + conventional treatment	NR	90	②③	NR
			C: 41/19						
Li et al. 2020	35/35	38.5 ± 4.1/35.2 ± 3.9	I: 13/22	Sacubitril/valsartan plus QLQX 1.2 g, tid + conventional treatment	Sacubitril/valsartan + conventional treatment	NR	56	①②③⑤	Scientific Research Program of Hebei Traditional Chinese Medicine Administration (No. 2018252)
			C: 18/17						
Li et al. 2021	54/53	60.17 ± 2.32/60.22 ± 2.5	I: 34/20	Sacubitril/valsartan plus QLQX 1.2 g, tid + conventional treatment	Sacubitril/valsartan + conventional treatment	NR	84	①②③④⑤	Scientific Research Project of Sichuan Medical Association (No. SHD14-21)
			C: 33/20						
Lin et al. 2021	41/42	62.42 ± 7.96	41/42	Sacubitril/valsartan plus QLQX 1.2 g, tid + conventional treatment	Sacubitril/valsartan + conventional treatment	NR	84	①②⑤	Quanzhou Science and Technology Project of Fujian Province (No. 2018N103s)
Liu 2020	38/42	71.52 ± 6.46/71.58 ± 6.39	I: 30/8	Sacubitril/valsartan plus QLQX 1.2 g, tid + conventional treatment	Sacubitril/valsartan + conventional treatment	NR	30	②③④	NR
			C: 31/11						
Ma et al. 2021	56/56	65.45 ± 10.51/66.17 ± 9.79	I: 30/26	Sacubitril/valsartan plus QLQX 1.2 g, tid + conventional treatment	Sacubitril/valsartan + conventional treatment	NR	84	①②③⑤	NR
			C: 32/24						
Qu 2020	34/34	65.34 ± 3.53/65.26 ± 3.46	I: 18/16	Sacubitril/valsartan plus QLQX 1.2 g, tid + conventional treatment	Sacubitril/valsartan + conventional treatment	NR	30	①②③⑤	NR
			C: 20/14						
Ren 2021	24/23	65 ± 4	I: 13/11	Sacubitril/valsartan plus QLQX 1.2 g, tid + conventional treatment	Sacubitril/valsartan + conventional treatment	NR	90	①②③④	Datong key Research and Development Program of Shanxi Province (No. 2020063)
			C: 14/9						
Shi 2018	38/38	71.5 ± 4.8/72.3 ± 5.3	I: 28/10	Sacubitril/valsartan plus QLQX 1.2 g, tid + conventional treatment	Sacubitril/valsartan + conventional treatment	NR	28	①②④⑤	NR
			C: 26/12						
Su et al.2020	37/37	59.06 ± 8.52/58.39 ± 8.06	I: 22/15	Sacubitril/valsartan plus QLQX 1.2 g, tid + conventional treatment	Sacubitril/valsartan + conventional treatment	NR	30	①②⑤	Zhanjiang Financial Fund Science and Technology Special Competitive Allocation Project (No. 2017A01029)
			C: 21/16						
Wang et al. 2021	40/40	72.3 ± 5.3	55/25	Sacubitril/valsartan plus QLQX 1.2 g, tid + conventional treatment	Sacubitril/valsartan + conventional treatment	NR	28	①②④	NR
Wang 2019	57/57	63.74 ± 5.7	63/51	Sacubitril/valsartan plus QLQX 1.2 g, tid + conventional treatment	Sacubitril/valsartan + conventional treatment	NR	30	①②④⑤	NR
Xu 2020	50/50	53.79 ± 4.46/54.13 ± 7.42	I: 22/28	Sacubitril/valsartan plus QLQX 0.6 g, tid + conventional treatment	Sacubitril/valsartan + conventional treatment	NR	84	①②③④	NR
			C: 27/23						
Yang 2021	43/43	62.64 ± 1.23/62.58 ± 1.17	I: 23/20	Sacubitril/valsartan plus QLQX 1.2 g, tid + conventional treatment	Sacubitril/valsartan + conventional treatment	NR	90	①⑤	NR
			C: 24/19						
Yao and li 2021	20/20	62.13 ± 3.16/61.28 ± 3.27	I: 11/9	Sacubitril/valsartan plus QLQX 1.2 g, tid + conventional treatment	Sacubitril/valsartan + conventional treatment	NR	30	①②④	NR
			C: 12/8						
Zhang 2020	44/44	76.5 ± 9.8/75.5 ± 9.4	I: 26/18	Sacubitril/valsartan plus QLQX 1.2 g, tid + conventional treatment	Sacubitril/valsartan + conventional treatment	NR	30	②④	NR
			C: 23/21						
Zhang 2021	41/41	64.86 ± 6.43/65.42 ± 6.19	I: 25/16	Sacubitril/valsartan plus QLQX 1.2 g, tid + conventional treatment	Sacubitril/valsartan + conventional treatment	NR	28	②③④	NR
			C: 22/19						

QLQX: Qiliqiangxin capsules; I, intervention group; C, control group; M, male; F, female; ① total effective rate; ② LVEF; ③ LVEDD; ④ 6-MWD; ⑤ Adverse events; NR: not report.

**Table 2:** Risk of bias of included studies.

Study	Random sequence generation (selection bias)	Allocation concealment (selection bias)	Blinding of participants and personnel (performance bias)	Blinding of outcome assessment (detection bias)	Incomplete outcome data (attrition bias)	Selective reporting (reporting bias)	Other bias
Dong and Du 2020	Low risk	Unclear risk	High risk	High risk	Low risk	Low risk	Unclear risk
Li et al. 2020	Low risk	Unclear risk	High risk	High risk	Low risk	Low risk	Unclear risk
Li et al. 2021	Low risk	Unclear risk	High risk	High risk	Low risk	Low risk	Unclear risk
Lin et al. 2021	Low risk	Unclear risk	High risk	High risk	Low risk	Low risk	Unclear risk
Liu 2020	High risk	Unclear risk	High risk	High risk	Low risk	Low risk	Unclear risk
Ma et al. 2021	High risk	Unclear risk	High risk	High risk	Low risk	Low risk	Unclear risk
Qu 2020	Low risk	Unclear risk	High risk	High risk	Low risk	Low risk	Unclear risk
Ren 2021	High risk	Unclear risk	High risk	High risk	Low risk	Low risk	Unclear risk
Shi 2018	High risk	Unclear risk	High risk	High risk	Low risk	Low risk	Unclear risk
Su et al.2020	High risk	Unclear risk	High risk	High risk	Low risk	Low risk	Unclear risk
Wang et al. 2021	High risk	Unclear risk	High risk	High risk	Low risk	Low risk	Unclear risk
Wang 2019	High risk	Unclear risk	High risk	High risk	Low risk	Low risk	Unclear risk
Xu 2020	Low risk	Unclear risk	High risk	High risk	Low risk	Low risk	Unclear risk
Yang 2021	Low risk	Unclear risk	High risk	High risk	Low risk	Low risk	Unclear risk
Yao and li 2021	Low risk	Unclear risk	High risk	High risk	Low risk	Low risk	Unclear risk
Zhang 2020	High risk	Unclear risk	High risk	High risk	Low risk	Low risk	Unclear risk
Zhang 2021	High risk	Unclear risk	High risk	High risk	Low risk	Low risk	Unclear risk

**Table 3:** The incidence rate of adverse effect.

Type	Study	The number of adverse effects	
		Experimental group	Control group
Dry cough	Li et al. 2020	0	1
Dizzy	Li et al. 2020	1	1
Rash	Li et al. 2020	1	1
Palpitation	Li et al. 2020	1	0
Hypotension	Ma et al. 2021	1	1
Angioedema	Ma et al. 2021	1	1
Gastrointestinal reaction	Ma et al. 2021	1	2
Potassium anomaly	Ma et al. 2021	0	1
Headache	Qu 2020	2	1
Nausea	Qu 2020, Yang 2021	3	5
Vomiting	Qu 2020, Yang 2021	1	3
Total event	—	12/358	17/358
Incidence rate	—	3.4%	4.7%

## Data Availability

The data used to support the findings of this study are included within the article.

## Abbreviations

CHF: Chronic heart failure
QLQX: Qiliqiangxin
LVEF: Left ventricular ejection fraction
LVEDD: Left ventricular end-diastolic dimension
6-MWD: Six-minutes walking distance
RCTs: Randomized controlled trials
CI: Confidence interval
MD: Mean difference
RR: Relative risk
PRISMA: Preferred Reporting Items for Systematic Reviews and Meta-Analyses.

## Supplementary Materials

Supplementary Materials Supplementary Material S1 PRISMA 2020 checklist.
